# Overview and Methods for the Youth Risk Behavior Surveillance System — United States, 2025

**DOI:** 10.15585/mmwr.su7501a1

**Published:** 2026-10-15

**Authors:** Jonetta J. Mpofu, Izraelle I. McKinnon, Argelia Figueroa, Zachary Myles, Shari Shanklin, Emiko Petrosky, Simone C. Gray, Ryan Saelee, Sandra J. Kiplagat, David Chyen, Jelonia T. Rumph, Malaika Washington, Cecily Mbaka, Cyprian Wejnert, Joana Lively, Barbara Queen, Alice Roberts

**Affiliations:** ^1^Division of Adolescent and School Health, National Center for Chronic Disease Prevention and Health Promotion, CDC, Atlanta, Georgia; ^2^U.S. Public Health Service, Rockville, Maryland; ^3^Westat, Bethesda, Maryland; ^4^ICF International, Rockville, Maryland

## Abstract

The Youth Risk Behavior Surveillance System (YRBSS) is the largest and most comprehensive source of data on adolescent health behaviors and experiences in the United States. Established in 1991, YRBSS includes a nationally representative Youth Risk Behavior Survey (YRBS) and separate site-level YRBSs conducted by states, tribal governments, territories, and local school districts. This overview report describes 2025 YRBSS survey methodology, including sampling, data collection, response rates, data processing, weighting, and analytic methods. The 2025 national survey response rates and student demographic characteristics are also included, along with the site-level participation map. During 2025, in addition to the national YRBS, 64 sites across the United States (including 40 states, one tribal government, one territory, and 22 local school districts) administered site-level YRBSs to high school students in their jurisdictions. YRBSS continues to capitalize on technological advances to make survey administration and data collection more efficient. The 2025 national YRBS was administered electronically using tablets for the second consecutive cycle. For the first time, approximately two thirds of participating sites also administered surveys electronically, representing a major milestone in the system’s transition toward electronic data collection. The increase in the number of sites opting to conduct electronic data collection opens new pathways for data modernization at the local level, including reducing the amount of work required for survey administration and time for data processing, enabling real-time logic checks, and allowing sites to more easily tailor questionnaires to jurisdictional needs. Although low participation and declining response rates remain challenging, the flexibility, reach, and adaptability of YRBSS support its continued role as a primary source of data on adolescent health. Continued participation in and promotion of the YRBSS by state, local, community, and school- and youth-focused professional organizations are essential to sustaining YRBSS participation and surveillance capacity.

## Introduction

The Youth Risk Behavior Surveillance System (YRBSS) is the leading source of data on adolescent health behaviors and experiences in the United States, with data from approximately 5 million youths who have participated at the national and local levels throughout the system’s more than 35-year history. Established in 1991, the YRBSS is comprised of a national school-based Youth Risk Behavior Survey (YRBS) conducted by CDC, alongside a network of state, territorial, tribal, and local YRBSs administered by health and education agencies. The structure of the YRBSS enables surveillance of a broad range of established and emerging adolescent health behaviors and experiences across national, state, and local levels and monitoring of changes in student health and well-being over time.

Adolescent health behaviors and outcomes are some of the most important predictors of adult health (*1*–*3*). Recent national data on adolescent health highlight several areas of continued public health concern, including poor mental health, increases in sedentary behavior and obesity, and decreases in healthy dietary, physical activity, and sleep behaviors (*4*–*7*). Developing effective prevention and intervention efforts to address these trends requires accurate, high-quality measurement of adolescent health behaviors and experiences. Furthermore, adolescent health behaviors and experiences can change over time and increasingly occur within rapidly changing digital environments, including social media and artificial intelligence (AI) technologies (*8*–*10*), underscoring the need for high-quality data systems that can respond to these shifts.

The YRBSS continually evolves to monitor adolescent health and well-being as adolescents navigate changing social and physical environments. In recent years, YRBSS has included questions on emerging health concerns affecting adolescents, such as indicators of mental health, social media, vaping, school connectedness, and parental monitoring, reflecting the ability of the system to adapt to current and anticipated youth health needs. However, the ability of YRBSS to track adolescent health behaviors effectively requires widespread school and student participation. Continued engagement from state, local, community, and national school-based and youth-focused professional organizations remains essential to maintaining the YRBSS as a robust source of data on key adolescent health behaviors and experiences.

This report details the 2025 YRBSS methodology, including sampling, data collection, data processing, weighting, and analytic procedures used across the system at the national level and at state and local levels. The remaining five reports in this supplement highlight the 2025 national data on critical adolescent health topics, including nutrition and physical activity, cardiovascular health, unmet need for mental health services, binge eating, and urban-rural differences in substance use. The report on cardiovascular health incorporates a novel use of YRBS data to derive a composite score for cardiovascular health aligned with the American Heart Association’s Life’s Essential 8 framework, with implications for chronic disease prevention beginning in adolescence. Two of the reports introduce emerging adolescent health topics by providing the first YRBS national estimates of binge eating and receipt of mental health services among high school students. Although findings from state and local survey data are excluded from this publication, these data are publicly available through CDC’s YRBSS web-based data dissemination tools, including YRBS Explorer (https://yrbs-explorer.services.cdc.gov), Youth Online (https://youthonline.cdc.gov), and the YRBS Analysis Tool (https://yrbs-analysis.cdc.gov).

## National YRBS Methodology

### Overview

The national YRBS is administered biennially during the spring semester of odd-numbered years among a nationally representative sample of students from public and private schools, including parochial schools, in grades 9–12 in the 50 U.S. states and the District of Columbia. Biennial administration enables CDC to assess temporal changes in behaviors among the U.S. high school population while adapting the instrument to identify emerging adolescent health behaviors and experiences affecting high school students. In addition, the national YRBS provides comparable data across survey years, enabling state and local jurisdictions conducting their own YRBS to demonstrate how behaviors and experiences of youths in their jurisdictions compare with those at the national level.

### Questionnaire

The national YRBS questionnaire uses single-item measures to monitor and describe a wide variety of health behaviors and experiences. The 2025 questionnaire contained 106 questions. Of these, 86 were included in the standard questionnaire used as a baseline by state and local jurisdictions; the remaining 20 addressed high-priority topic areas for CDC and other partners. The standard questionnaire is periodically revised to include new questions that assess emerging problems and evolving health behaviors among high school students. Proposed revisions are reviewed by subject matter experts representing CDC, academia, other federal agencies, nongovernmental organizations, and YRBS coordinators from supported agencies and jurisdictions nationwide. Proposed new and modified items undergo cognitive testing with high school students to evaluate comprehension and interpretation before being included in the questionnaire. To maintain the 90-item limit on the standard questionnaire, a “one-in, one-out” rule is applied whereby items are removed if they are no longer applicable, exhibit low prevalence, or are frequently omitted by participating sites. Proposed changes are submitted to YRBS coordinators for a vote, and additions, modifications, and deletions are finalized by simple majority. To support accessibility and participation, the national YRBS questionnaire was administered in both English and Spanish.

Most survey questions used a multiple-choice structure with up to eight mutually exclusive options, allowing for only one response per question. Exceptions to this format were questions assessing height, weight, and race. A test-retest study of the 2021 YRBS survey questions demonstrated substantial reliability among these questions (*11*). The wording of each question, including recall periods, response options, and operational definitions for each variable, is available in the 2025 YRBS questionnaire and data user’s guide. (YRBSS data and documentation are available at https://www.cdc.gov/yrbs/data/index.html.)

For the second consecutive YRBS administration, the 2025 national YRBS was self-administered electronically using tablets. Electronic data collection enhanced overall data integrity by incorporating real-time logic checks. For example, the questionnaire was programmed to prompt students to correct invalid or out-of-range responses for height and weight, thereby reducing the need for postcollection data cleaning. The national YRBS underwent the Office of Management and Budget (OMB) review process, and Paperwork Reduction Act determinations for these projects are available (https://www.reginfo.gov/public/do/PRAOMBHistory?ombControlNumber=0920-0493).

### Sampling

The sampling frame for the 2025 national YRBS included regular public (including charter) schools and private (including parochial) schools with students in grades 9–12 in the 50 U.S. states and the District of Columbia. Exclusion criteria included alternative, special education, U.S. Department of Defense, Bureau of Indian Education, and vocational schools serving students who also attended another school. Schools with 40 or fewer students enrolled in grades 9–12 (combined) were excluded. The sampling frame was constructed from data files obtained from MDR (formerly Market Data Retrieval) and the National Center for Education Statistics (NCES). NCES data sources included the Common Core of Data (CCD) (https://nces.ed.gov/ccd/) for public schools and the Private School Universe Survey (PSS) (https://nces.ed.gov/surveys/pss/) for private schools.

A three-stage cluster sampling design was used to produce a nationally representative sample of students in grades 9–12 attending public and private schools. In the first stage, the sampling frame comprised 1,072 primary sampling units (PSUs), consisting of entire counties, groups of smaller adjacent counties, or parts of larger counties. PSUs were categorized into 16 strata according to metropolitan statistical area status (i.e., urban or nonurban) and the percentages of Black or African American (Black) and Hispanic or Latino (Hispanic) students. Of the 1,072 PSUs, 70 were selected with probability proportional to overall school enrollment size.

In the second stage, secondary sampling units (SSUs) within the 70 sampled PSUs were defined as a physical school containing grades 9–12 or a unit created by combining nearby schools to provide all four grades. A total of 210 SSUs were selected with probability proportional to school enrollment size. An additional 20 small SSUs were selected from a random subsample of the 70 PSUs to improve coverage of students attending small schools, resulting in 230 SSUs corresponding to 230 physical schools.

In the third stage, one or two classrooms in each of grades 9–12 were randomly selected within participating schools from a required subject (e.g., English or social studies) or a required period (e.g., homeroom or second period). All students in sampled classes who could independently complete the questionnaire were eligible to participate. Schools, classes, and students that declined participation were not replaced.

### Data Collection Procedures

This study was reviewed and approved by institutional review boards at CDC and ICF, the survey contractor, and was conducted consistent with applicable federal law and CDC policy.* Parental permission procedures were followed before survey administration. Survey procedures for electronic administration were designed to protect students’ privacy and allow for anonymous and voluntary participation. During electronic administration, students completed the survey individually using tablets programmed with the survey instrument and no personally identifying information was collected. The tablets were not connected to the internet. Trained data collectors visited each participating school to distribute the tablets to the students and collect them after survey completion; at the end of each day, data collectors uploaded all locally stored data from the tablets to a secure data collection platform. These procedures were intended to maintain privacy and anonymity while students completed the survey in the classroom.

The 2025 national YRBS was administered during the spring semester. Students completed the self-administered questionnaire during one class period. Students typically completed the tablet-based questionnaire in under 20 minutes. Because data became available for processing immediately after the daily uploads, CDC was able to monitor data collection progress in near real-time. In addition, a web-based version of the questionnaire was provided for schools that preferred online administration and for students who were absent on the day of data collection or who were late in submitting their consent forms. A total of 827 surveys were completed using this web-based platform.

### Response Rates and Data Processing

Of the 230 physical schools selected for the national sample, 108 participated, yielding a school response rate of 47.0%. Among sampled students in participating schools, the student response rate was 65.2%, resulting in an overall response rate (i.e., [student response rate] × [school response rate]) of 30.6%.

From these 108 schools, 11,097 students completed questionnaires. The data were cleaned to improve the quality and internal consistency of the final dataset. Responses were checked for valid response ranges, biologically plausible height and weight values, and logical consistency across related questions. Invalid responses, including responses outside the allowable range or logically inconsistent responses across related items, were set to missing. No statistical imputation was performed for missing data. Questionnaires failed quality control if fewer than 20 responses remained after editing or if they exhibited potential straightlining (the same response option was selected for 15 or more consecutive questions). Among the 11,097 completed questionnaires, 387 failed quality control and were excluded from analysis, resulting in 10,710 usable questionnaires.

The 2025 YRBSS adopted the updated 2024 OMB standards for maintaining, collecting, and presenting race and ethnicity data (https://www.federalregister.gov/documents/2024/03/29/2024-06469/revisions-to-ombs-statistical-policy-directive-no-15-standards-for-maintaining-collecting-and). Data were collected using a single combined question (“What is your race and/or ethnicity?”), allowing students to select multiple options across seven categories: 1) American Indian or Alaska Native, 2) Asian, 3) Black or African American (Black), 4) Hispanic or Latino (Hispanic), 5) Middle Eastern or North African (ME/NA), 6) Native Hawaiian or Pacific Islander, or 7) White. For the analyses presented in this supplement, students who selected the Hispanic option were categorized exclusively as Hispanic, regardless of any additional racial categories selected (e.g., a student selecting both Hispanic and Black or White was classified as Hispanic). Consequently, all other single-race categories represent non-Hispanic students. Non-Hispanic students who selected two or more racial categories were classified as multiracial. Race and ethnicity were coded as missing for students who left this question blank.

### Weighting

Weights were applied to the final 2025 national YRBS sample to produce a nationally representative sample of students from public and private schools, including parochial schools, in grades 9–12. The final survey weight incorporated the probability of selection at each stage of sampling and separate adjustments for school- and student-level nonresponse. The final weight was calculated as the product of the school base weight, student base weight, school nonresponse adjustment factor, student nonresponse adjustment factor, and poststratification adjustment factor. Thus, unequal probabilities of selection were incorporated into the base weights, whereas nonresponse adjustments accounted for observed participation at the school and student levels. The weights were then scaled so that the sum of the weights equaled the total sample size and the weighted distributions of students by sex, race and ethnicity, and grade matched national population proportions from the 2021–2022 CCD and 2021–2022 PSS.

## Analytic Methods

Findings presented in this supplement are derived from analytic procedures similar to those described in this overview report. For more information about the detailed analyses presented in other reports in this supplement (e.g., variables analyzed, custom measures, and data years), see the Methods section in each individual report. Missing data, whether resulting from questionnaire design or respondent-level missing data (e.g., skipped questions, out-of-range responses, or logical inconsistencies), were not statistically imputed; analyses used available valid responses for each measure, resulting in variable-specific analytic denominators.

All statistical analyses used SAS-callable SUDAAN (version 11.0.3 or 11.0.4; RTI International) to account for the complex sampling design and weighting. Prevalence estimates and CIs were computed for variables used in each report. Prevalence estimates with denominators <30 were considered statistically unreliable and therefore were suppressed. Differences in prevalence by demographic characteristics (e.g., sex, race and ethnicity, and grade) were assessed using design-adjusted *t* tests for specified pairwise comparisons. Domain analysis was used to calculate standard errors, CIs, and p-values appropriately in the presence of variable-specific missing data. Prevalence ratios were estimated using logistic regression with predicted marginals. All prevalence estimates and measures of association used Taylor series linearization. Differences were considered statistically significant at p<0.05; prevalence ratios were considered statistically significant when 95% CIs did not cross the null hypothesis value of 1.0.

For temporal trend analyses reported in YRBSS web-based data dissemination tools, logistic regression was used to assess linear and quadratic changes in estimates while controlling for changes over time in sex, grade, and race and ethnicity. Quadratic trends were used as an initial test for departures from linearity, such as leveling off, acceleration, or changes in direction. Linear trends were assessed to evaluate overall increases or decreases over time. Linear and quadratic time variables were treated as continuous and coded using orthogonal coefficients calculated with PROC IML in SAS (version 9.4; SAS Institute). A minimum of 3 survey years was required to assess linear trends and six survey years to assess quadratic trends; separate models were used for each. When a significant quadratic trend was identified, Joinpoint (version 5.02; National Cancer Institute) was used to identify the year when the trend changed, and regression models were then used to characterize linear trends before and after the identified change point. Cubic and higher-order trends were not assessed. A p-value <0.05 associated with a regression coefficient was considered statistically significant.

For analyses of 2-year changes in reports in this supplement and YRBSS web-based data dissemination tools, prevalence estimates from 2023 and 2025 were compared using design-adjusted *t* tests for behaviors, experiences, or conditions assessed with identically worded questions in both survey years. Prevalence estimates were considered statistically different if the *t* test p-value was <0.05.

## State, Tribal, Territorial, and Local School District YRBS Methodology

### Overview

Biennial administration of YRBS by state, tribal, territorial, and local education and health agencies (sites) allows them to monitor health behaviors, experiences, and conditions among their respective high school populations. Survey data are comparable across years within jurisdictions and can support comparisons of state and local estimates with national estimates. However, differences among the populations represented should be considered when making such comparisons: the national YRBS represents students attending public and private (including parochial) schools, whereas site-level YRBSs generally represent students attending public schools within their jurisdictions. During the 2025 cycle, 64 sites conducted a YRBS, including 40 states, one tribal government, one territory, and 22 local school districts (Figures 1 and 2). Survey administration was staggered: four sites conducted surveys in fall 2024, 38 in spring 2025, and 22 in fall 2025. The survey was self-administered anonymously within a single class period, taking ≤45 minutes to complete by paper and pencil and <20 minutes for electronic administration. Each site followed jurisdictional requirements for institutional review board approval. Data collection, processing, and analytic methods generally followed those described for the national YRBS; however, in 2025, a total of 42 sites collected data electronically using computers, smartphones, or tablets, and 22 used paper-and-pencil questionnaires and scannable answer sheets.

**FIGURE 1:**
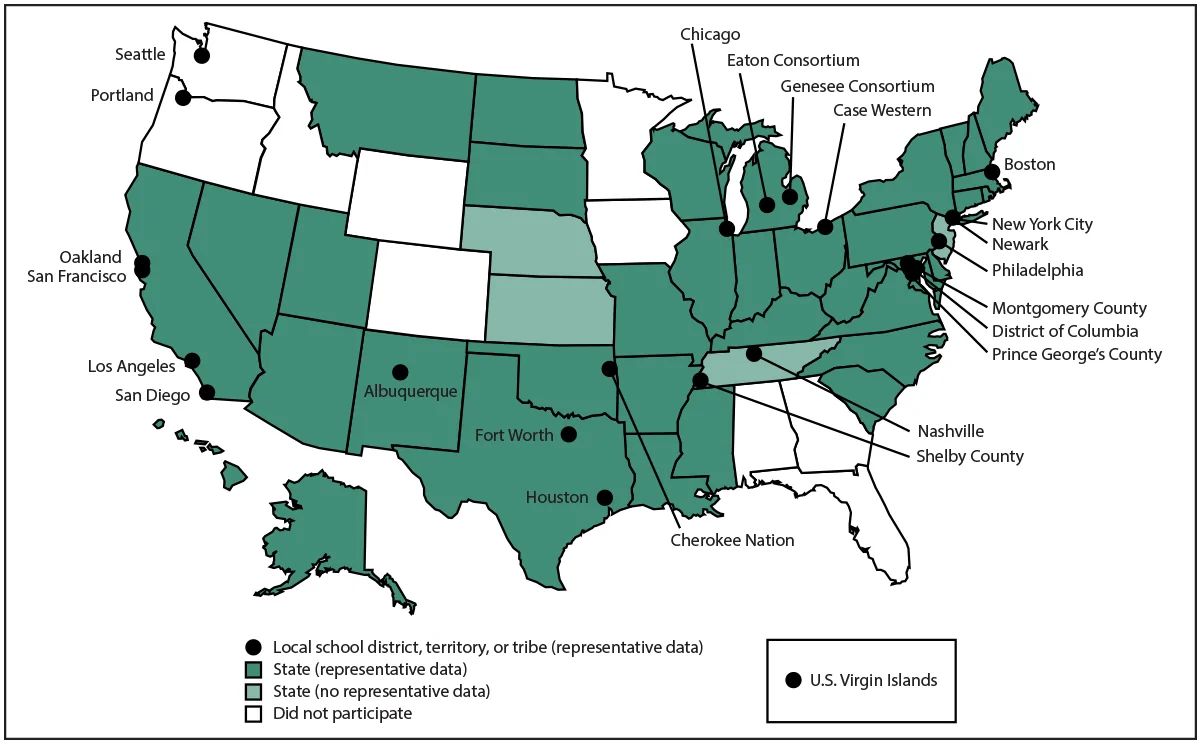
States, tribal governments, territories, and local school districts that conducted site-level Youth Risk Behavior Surveys — selected U.S. sites, 2025

**FIGURE 2:**
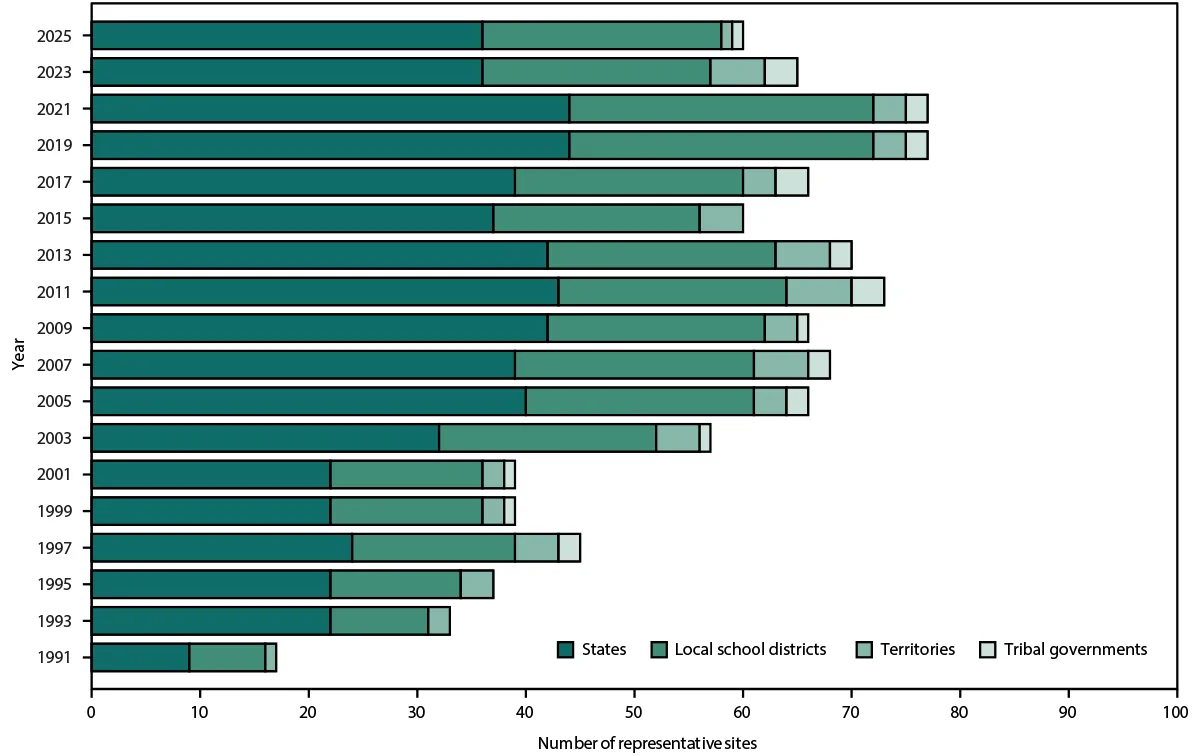
Number of states, tribal governments, territories, and local school districts that conducted site-level Youth Risk Behavior Surveys with representative data (independent of the national survey), by year of survey — Youth Risk Behavior Survey, selected U.S. sites, 1991–2025

### Questionnaires

The 2025 standard YRBS questionnaire contained 86 questions and served as the baseline instrument for site-level YRBS questionnaires. Sites were allowed to add or delete questions but were required to retain at least 57 of the questions on the standard questionnaire, including all demographic questions. This flexibility allowed YRBS sites and other state and local partners the opportunity to tailor the instrument to meet local data needs and programmatic priorities.

### Sampling

The sampling frame for the 2025 standard YRBS included regular public schools with students in grades 9–12. A two-stage cluster sampling design was used to produce representative samples of high school students in participating jurisdictions. In the first stage, public schools with any of grades 9–12 were sampled with probability proportional to school enrollment size. In the second stage, classes from either a required subject (e.g., English or social studies) or a required period (e.g., homeroom or second period) were randomly sampled within participating schools. All students in sampled classes who could independently complete the survey were eligible to participate. Exceptions to this two-stage design occurred in two sites (Vermont and the District of Columbia) that conducted a complete census of all eligible schools and students.

To reduce respondent effort, students in schools selected for both the national and site-level samples participated in only one YRBS. In these selected schools, either the national or the site-specific questionnaire was administered, and data from identical questions were subsequently shared between sites. Because site-specific questionnaires differ from the national questionnaire, students in schools sampled for the national and site-level surveys might not have been asked all national YRBS questions. Therefore, the total denominator of students answering each question in the national dataset varies.

## Nonresponse Bias Analyses, Weighting, and Response Rates

Site-level datasets were cleaned and edited for inconsistencies. Missing data were not statistically imputed. A questionnaire failed quality control when fewer than 20 responses remained after editing or when it contained the same answer to 15 or more consecutive questions. CDC conducted nonresponse bias analyses (NRBAs) for all sites with response rates ≥10% to determine whether data for each site could be weighted to be representative of its jurisdiction. These analyses compared responding and nonresponding schools on school enrollment size (small, medium, or large), a measure of the school’s poverty level (usually the percentage of students eligible for free or reduced-price lunch), and locale type (city, suburban, town, or rural). Analyses also compared responding and nonresponding students by grade. Weighted sample distributions were then compared with population distributions by grade, sex, and race and ethnicity. If limited statistically significant differences between comparison groups were found, data were weighted to be representative of their respective populations.

Site-level survey weights were constructed using the same general weighting framework described for the national YRBS, including school and student base weights, school and student nonresponse adjustments, and poststratification adjustments. Site-level weights were calibrated to the student population within each jurisdiction so that the weighted count equaled the corresponding student population. A total of 36 states, one tribal government, one territory, and 22 local school districts had representative (weighted) data in 2025 (Figures 1 and 2). In two states, weighted estimates were representative of all students in grades 9–12 attending regular public schools.

## 2025 National YRBS Response Rates, Nonresponse Analyses, and Demographic Characteristics

The overall response rate continued to decline, reaching 30.6% for the 2025 national YRBS cycle. Although student participation (65.2%) remained comparable to previous cycles (Figure 3), the school response rate (47.0%) declined slightly from 2023 and was substantially lower than historical levels. The student response rate reflects participation among sampled students and does not separately distinguish sources of student nonresponse, including absence, lack of parental permission, or student refusal.

**FIGURE 3:**
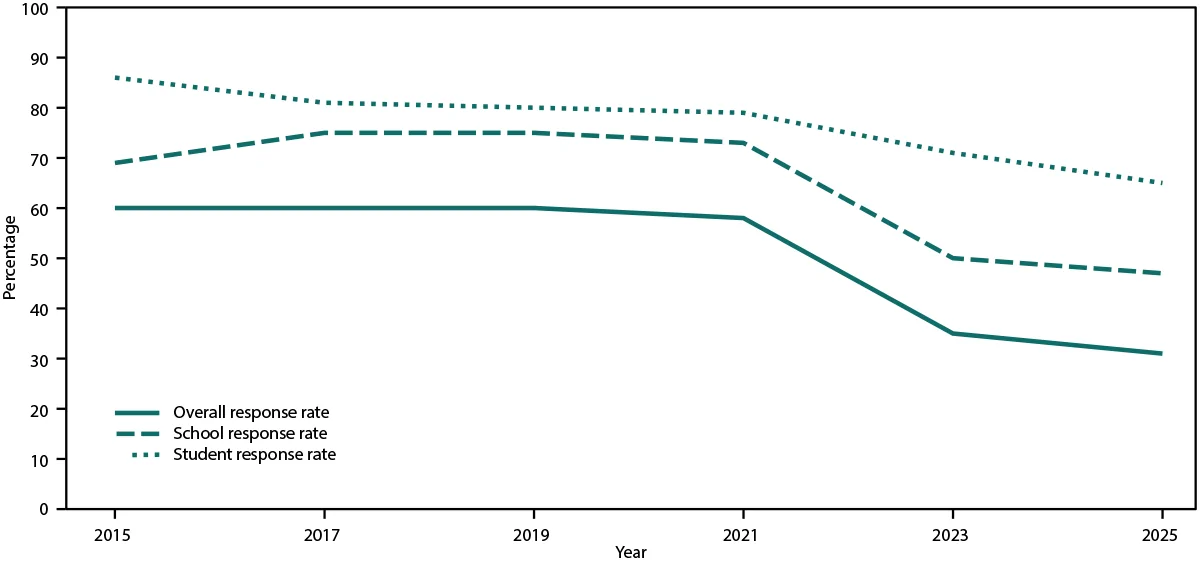
Overall, school, and student response rates for the Youth Risk Behavior Survey — United States, 2015–2025

Overall response rates in YRBSS have decreased steadily since 2011 and were <60% in 2021 during the COVID-19 pandemic. However, survey methodology research indicates that a high response rate does not necessarily indicate an unbiased sample, nor does a low response rate inherently indicate greater bias (*12*). For the 2025 national YRBS, nonresponse analyses included bivariate and multivariable analyses of school and student-level characteristics associated with participation. Bivariate analyses identified significant differences between participating and nonparticipating schools by school type (public versus nonpublic), locale status (city, suburb, town, rural), city (versus noncity), lunch program participation, Title I expenditures, majority White status (no versus yes), and poverty rate. In a multivariable logistic regression model including these characteristics, school type, city status, and poverty level remained significant predictors of school participation: nonpublic schools and schools below the median poverty level were less likely to participate, whereas schools in cities were more likely to participate. Weighting adjustments incorporated these characteristics to reduce potential nonresponse bias (ICF, unpublished data, 2026).

These nonresponse analyses evaluate whether characteristics of responding and nonresponding schools and students differ but do not assess nonresponse bias separately for each health behavior or experience measured by YRBS. Because patterns of response and missing data can vary across survey items, the potential for item-specific nonresponse bias should be considered when interpreting individual estimates.

The 2025 national YRBS data were weighted to match national population proportions. After weighting, approximately half of students were male (51.7%), and percentages of students by grade were as follows: grade 9 (27.3%), grade 10 (25.4%), grade 11 (24.0%), and grade 12 (23.3%) (Table). In addition, 47.8% of students were White, followed by Hispanic (27.0%), Black (11.7%), multiracial (5.9%), Asian (5.4%), ME/NA (1.2%), American Indian or Alaska Native (0.6%), and Native Hawaiian or Pacific Islander (0.3%).

**TABLE:** Student demographic characteristics — national Youth Risk Behavior Survey, United States, 2025

Characteristic	No. (%)
Student sample size*	**10,710 (100)**
**Sex^†^**	
Female	5,235 (48.3)
Male	5,435 (51.7)
**Race and ethnicity^§,¶^**	
American Indian or Alaska Native	78 (0.6)
Asian	517 (5.4)
Black or African American	2,169 (11.7)
Hispanic or Latino	3,353 (27.0)
Middle Eastern or North African	104 (1.2)
Native Hawaiian or Pacific Islander	33 (0.3)
White	3,603 (47.8)
Multiracial	760 (5.9)
Grade**	
9	3,086 (27.3)
10	2,763 (25.4)
11	2,610 (24.0)
12	2,209 (23.3)

* Among the 11,097 completed questionnaires, 387 failed quality control and were excluded from analysis, resulting in 10,710 usable questionnaires.

^†^ Does not include 40 students who did not indicate sex.

^§^ Persons of Hispanic or Latino (Hispanic) origin might be of any race but are categorized as Hispanic; all racial groups are non-Hispanic.

^¶^ Does not include 93 students who did not indicate race, ethnicity, or both.

** Does not include 19 students who responded, "ungraded or other grade" and 23 students who did not indicate a grade.

## Data Availability and Dissemination

National and site-level YRBS data (2019–2025) are available in a combined dataset from the YRBSS data and documentation website (https://www.cdc.gov/yrbs/data/index.html), as are additional resources, including data documentation and analysis guides. Data are available in both Access and ASCII formats, and SAS and SPSS programs are provided for converting the ASCII data into SAS and SPSS datasets. Variables are standardized to facilitate trend analyses and for combining data. YRBSS data also are available online via three web-based data dissemination tools: Youth Online, YRBS Analysis Tool, and YRBS Explorer. Youth Online allows point-and-click data analysis and creation of customized tables, graphs, maps, and fact sheets (https://youthonline.cdc.gov). Youth Online also performs statistical tests by health topic and filters and sorts data by demographic characteristic. The YRBS Analysis Tool allows real-time analysis of YRBS data and generates frequencies, cross-tabulations, and stratified results (https://yrbs-analysis.cdc.gov). YRBS Explorer is an application featuring options to view and compare national, state, and local data via tables and graphs (https://yrbs-explorer.services.cdc.gov).

Availability of site data depends on survey participation, data quality, and data-sharing policies. Information about YRBSS data is available on the participation maps and history website (https://www.cdc.gov/yrbs/data/yrbs-participation.html). Site-level YRBS data collected during 1991–2025 are available through Youth Online (https://youthonline.cdc.gov/), the YRBS Analysis Tool (https://yrbs-analysis.cdc.gov), and YRBS Explorer (https://yrbs-explorer.services.cdc.gov).

## Discussion

The YRBSS is the largest and most comprehensive collection of national and local data on youth health behaviors and experiences in the U.S., serving as a foundational resource for understanding adolescent health behaviors and outcomes. Innovations in the 2025 YRBS included the introduction of new national-level survey questions on binge eating and mental health services and the adoption of the updated 2024 OMB standards for maintaining, collecting, and presenting race and ethnicity data using a single combined question. This update introduced the ME/NA race and ethnicity category to YRBSS data collection.

YRBSS continues to face challenges with declining response rates. The 2025 national YRBS overall response rate of 30.6% was the lowest in the survey’s history. Although weighting and nonresponse analyses are used to support use of the national estimates, declining participation at the state and local levels can reduce the number of jurisdictions with representative YRBS data available to schools, communities, and decision makers. Multiple factors might contribute to declining participation, including challenges obtaining district and school participation and parental permission, competing demands on instructional time, and resource constraints. The relative contributions of these factors to changes in YRBSS participation have not been systematically evaluated.

CDC has implemented several approaches intended to support YRBSS participation and data quality. Since 2021, CDC has conducted NRBAs for site-level YRBSs to determine whether data from sites with lower response rates can be weighted to represent their jurisdiction. This has allowed many sites with lower response rates to receive weighted data, which are of substantial benefit to states and local sites, staff members, and students who take time to participate in YRBS. The 2023 cycle also marked the full transition to electronic data collection for the national YRBS, reducing survey administration effort and data processing time; the percentage of sites electing electronic data collection increased from 47% in 2023 to 65% in 2025. Previous research comparing electronic and paper-and-pencil YRBS administration found that mode of administration did not meaningfully affect prevalence estimates, supporting comparability across administration modes (*13*). Maintaining comparability across survey cycles is essential to monitoring trends over time. Although core sampling, weighting, and analytic methods are designed to support temporal comparisons, YRBSS also evolves in response to changes in technology, participation, and data collection needs. Continued methods research is important to determine whether such changes affect estimates or interpretation of trends.

Additional efforts are needed to improve YRBSS participation. Research on school surveys suggests that minimizing respondent fatigue, coordinating sampling, and providing incentives can support participation (*14*). Several recent and planned YRBSS enhancements align with these approaches. Electronic data collection is intended to reduce administrative effort and coordination of national and site-level samples reduces duplication for schools selected for both surveys. Planned increases in school and teacher incentives and provision of school-level data to schools participating in the 2027 national YRBS are intended to support participation and demonstrate its value. Partner organizations can also serve as trusted collaborators by communicating the value of YRBSS data and participation to state and local health and education agencies, school districts, and schools. The effects of these individual strategies on YRBSS response rates have not been established, underscoring the importance of continued evaluation. Findings from reports in this supplement using national YRBS data are aligned with longstanding public health priorities and initiatives and also support the need for improvements in adolescent health. For example, the report on dietary and physical activity behaviors suggests that, despite certain improvements since 2023, the prevalence of students engaging in healthy dietary and physical activity behaviors, including behaviors aligned with the recently released 2025–2030 Dietary Guidelines for Americans, remains low (*15*,*16*). As chronic disease prevention among adolescents receives increasing national attention, another report uses YRBS data to estimate overall cardiovascular health among high school students using five of the eight metrics aligned with the American Heart Association’s Life’s Essential 8 framework (*17*). This novel application of YRBS data suggests that only one in five students had high, or favorable, cardiovascular health; among the five metrics assessed using YRBS data, diet, physical activity, and sleep health had the least favorable scores (*18*). In response to persistent concerns about adolescent mental health, a new YRBS question assessing receipt of mental health counseling or therapy was added in 2025. Findings from this report indicate that among students experiencing persistent feelings of sadness or hopelessness, approximately one in three reported an unmet need for counseling or therapy because of barriers such as cost or not knowing how or where to seek help (*19*). In collaboration with partners in the field of eating disorder prevention and research, YRBS also included a new item assessing binge eating for the first time in 2025. Approximately 17% of high school students reported binge eating on ≥3 days during the past 30 days, and prevalence was higher among students who used social media hourly or more and among those reporting indicators of poor mental health and suicide risk (*20*). Finally, recent enhancements to the surveillance system have enabled linkage of YRBS data with school-level information, allowing for examination of substance use behaviors by urban-rural classification using the national YRBS. Findings from this report indicate a higher prevalence of tobacco and nicotine product use among students in rural public schools, suggesting potential opportunities for tailored prevention strategies in rural schools and communities (*21*). Collectively, these reports demonstrate the usefulness of YRBSS for examining evolving public health priorities and generating actionable data to guide programs and interventions aimed at improving adolescent health.

## Limitations

Each report in this supplement includes limitations specific to its variables and analyses. In general, YRBSS findings are subject to at least five limitations. First, national YRBS estimates apply to students in grades 9–12 attending U.S. public and private (including parochial) schools; site-level estimates apply to the school populations represented by each participating jurisdiction. Homeschooled youths and youths not attending school are not represented. In 2022, approximately 5% of youths aged 14–17 years were not enrolled in school (https://nces.ed.gov/programs/digest/d23/tables/dt23_103.20.asp). Second, the 2025 national YRBS had an overall response rate of 30.6%, driven largely by a school response rate of 47.0%. Nonresponse analyses found that in a multivariable logistic regression model, school type, city, and poverty level were significant predictors of school participation (ICF, unpublished data, 2026). Third, health-related behaviors and experiences are self-reported, and the extent of underreporting or overreporting cannot be determined. Most YRBS questions undergo cognitive testing to determine whether students understand and interpret the questions as intended, and a 2021 study found substantial test-retest reliability for most YRBS questions (*11*). However, cognitive testing and test-retest reliability do not establish the accuracy of self-reported behaviors and experiences. Fourth, students in schools selected for both the national and a site-specific sample were surveyed only once, often using the site-specific questionnaire. As a consequence, certain students contributing to the national dataset were not administered all national YRBS questions. Available data therefore varied across questions because of questionnaire design as well as respondent-level missing data. Finally, YRBSS surveys are cross-sectional and can identify associations but cannot establish causality; the surveillance system is descriptive and is not designed to determine definitive reasons underlying observed trends.

## Conclusion

YRBSS continues to provide data critical to guiding and supporting adolescent health and well-being in the United States. Despite persistent participation challenges, the system remains flexible and able to adapt and survey evolving adolescent health problems and data needs. YRBSS success depends on continued engagement from federal, state, and local partners, school and youth-focused organizations and the students and families who participate in the surveys nationwide.

This overview report describes 2025 YRBSS methods and procedures including those used to guide the analyses presented in this supplement. A full description of 2025 YRBSS findings and downloadable data from the national and site-specific surveys are available at https://www.cdc.gov/yrbs/data/index.html.
